# Generative AI technostress and learning engagement among Chinese university students: a moderated mediation model of academic adaptability and AI literacy

**DOI:** 10.3389/fpsyg.2026.1955418

**Published:** 2026-09-03

**Authors:** Tongyu Su

**Affiliations:** Anshan Normal University, Anshan, China

**Keywords:** academic adaptability, AI literacy, generative artificial intelligence, higher education, learning engagement, technostress

## Abstract

The increasing integration of generative artificial intelligence (GenAI) into higher education has altered how students access information, complete academic tasks, and participate in learning activities. Alongside these opportunities, GenAI introduces technology-related demands that may be associated with students’ learning engagement. This study examined the relationship between generative AI technostress and learning engagement, with academic adaptability examined as a statistical mediator and AI literacy as a moderator of this relationship. A quantitative cross-sectional research design was adopted. Using nonprobability convenience sampling, data were collected through an online self-report questionnaire administered via Wenjuanxing to undergraduate students with experience using GenAI for academic purposes at Northeast Normal University in China. A total of 529 valid responses were analyzed using structural equation modeling and bootstrap-based conditional process analysis. Generative AI technostress was negatively associated with academic adaptability and learning engagement, whereas academic adaptability was positively associated with learning engagement. A significant indirect association between generative AI technostress and learning engagement through academic adaptability was identified, while the direct association remained significant. AI literacy also moderated the negative association between generative AI technostress and academic adaptability, with a weaker negative association observed among students reporting higher AI literacy. The conditional indirect association through academic adaptability was stronger at lower levels of AI literacy. Taken together, the findings indicate that the relationship between generative AI technostress and learning engagement is associated with students’ capacity to adapt to changing academic demands and varies according to their level of AI literacy.

## 1 Introduction

Learning engagement is an important dimension of students’ participation in higher education and encompasses behavioral, cognitive, emotional, and social involvement in learning activities (Fredricks et al., 2004; Zhoc et al., 2019). As generative artificial intelligence becomes increasingly embedded in university learning, students’ engagement is developing within an environment where information access, academic assistance, feedback, writing support, and self-regulated learning are increasingly mediated by AI systems (Barrett and Pack, 2023; Xia et al., 2024). Applications such as ChatGPT, Gemini, and DeepSeek may therefore shape how students complete academic tasks, allocate attention, evaluate information, and regulate learning strategies (Crompton and Burke, 2023; Lim et al., 2023; Kasneci et al., 2023; Tlili et al., 2023). Understanding factors associated with learning engagement in such environments has consequently become increasingly relevant to higher education research.

Existing research on generative AI in education has largely examined perceived usefulness, technology acceptance, instructional applications, academic integrity, and ethical considerations (Chan and Hu, 2023; Lo, 2023). Although GenAI can support academic activities, its use also requires students to understand system capabilities and limitations, evaluate generated information, identify unreliable outputs, and determine appropriate forms of AI assistance (Chan, 2023; Delcker et al., 2024; Kasneci et al., 2023). Rapid technological development and variation in institutional expectations may further increase uncertainty regarding appropriate academic use (Walter, 2024; Stone, 2025), while evaluating AI-generated information may require additional cognitive resources (Hou et al., 2025).

These demands provide a basis for examining GenAI use through the concept of technostress. Technostress refers to psychological strain associated with technology-related demands that individuals perceive as difficult to manage with available resources (Ayyagari et al., 2011; Tarafdar et al., 2007). In educational settings, it has been associated with technological complexity, information overload, uncertainty, and continuing requirements for adjustment (Wang et al., 2020). Generative AI technostress shares these characteristics but also involves demands related to evaluating probabilistic outputs, identifying inaccuracies, and determining appropriate reliance on AI-generated information (Kasneci et al., 2023). Although technostress has been examined in technology-enhanced learning, its association with students’ adjustment and engagement in GenAI-mediated learning remains less fully established (Klimova and Pikhart, 2025).

The relationship between GenAI technostress and learning engagement may also involve students’ capacity to adjust to changing academic demands. The transactional theory of stress and coping suggests that responses to environmental demands depend partly on individuals’ appraisal and adjustment processes (Folkman, 2013). Academic adaptability captures students’ capacity to adjust their cognitive, behavioral, and emotional responses when academic circumstances are changing or uncertain (Martin, 2012), and has been associated with academic functioning and engagement (Martin et al., 2013; Collie et al., 2017). It may therefore help explain part of the association between GenAI technostress and learning engagement.

Students may also differ in the personal resources available to manage AI-related demands. AI literacy refers to the knowledge, skills, and critical understanding required to understand, use, and evaluate artificial intelligence appropriately (Long and Magerko, 2020; Wang et al., 2023). Students with stronger AI literacy may be better able to understand AI capabilities and limitations, evaluate outputs, and manage AI-supported learning activities (Ng et al., 2021; Bećirović et al., 2025). From the perspective of conservation of resources theory, AI literacy may therefore be regarded as a personal resource associated with how students respond to demanding AI-mediated learning conditions (Hobfoll, 1989).

Previous studies have provided evidence concerning GenAI use, technostress, academic adaptability, AI literacy, and learning engagement, but these lines of research remain relatively fragmented. Technostress has been associated with students’ motivation, learning behaviors, and psychological experiences (Wang et al., 2020; Frazier et al., 2019), academic adaptability has been positively associated with academic functioning and engagement (Martin et al., 2013; Collie et al., 2017), and AI literacy has been linked to students’ capacity to understand and manage AI systems (Ng et al., 2021; Wang et al., 2023; Bećirović et al., 2025). However, less is known about how these constructs operate together in GenAI-mediated learning. It remains unclear whether academic adaptability accounts for part of the association between generative AI technostress and learning engagement and whether this association varies according to students’ AI literacy.

The study examines whether generative AI technostress is associated with learning engagement directly and indirectly through academic adaptability, and whether AI literacy moderates the association between generative AI technostress and academic adaptability. The proposed moderated mediation model is examined using data from undergraduate students at Northeast Normal University in China.

The study contributes to research on GenAI-mediated learning in three respects. First, it focuses on learning engagement as an important correlate of GenAI technostress rather than emphasizing technology adoption or instructional utility. Second, it examines academic adaptability as a psychological adjustment mechanism that may account for part of the association between GenAI technostress and learning engagement. Third, it considers AI literacy as a personal resource associated with variation in students’ adjustment to GenAI-related demands.

## 2 Theoretical background and hypotheses development

This study integrates the transactional theory of stress and coping and conservation of resources theory to explain the proposed moderated mediation model. The transactional theory of stress and coping emphasizes that individuals’ responses to environmental demands depend on how those demands are appraised and managed (Folkman, 2013). In GenAI-mediated learning, students may experience technostress when AI-related academic demands are perceived as difficult to manage. Academic adaptability reflects students’ capacity to adjust their cognitive, behavioral, and emotional responses to such changing or uncertain academic conditions (Martin, 2012; Martin et al., 2013), and therefore provides a basis for understanding the indirect association between generative AI technostress and learning engagement.

Conservation of resources theory complements this process-oriented perspective by emphasizing the role of personal resources in managing demanding situations (Hobfoll, 1989; Hobfoll et al., 2018). AI literacy represents a relevant personal resource because it involves the knowledge and competencies required to understand, use, evaluate, and appropriately manage AI technologies (Wang et al., 2023). Students with higher AI literacy may therefore be better equipped to manage GenAI-related demands, which may be reflected in a weaker negative association between technostress and academic adaptability.

Together, the two theories provide complementary explanations for the proposed model. The transactional theory of stress and coping explains the adjustment pathway linking generative AI technostress, academic adaptability, and learning engagement, while conservation of resources theory explains why this pathway may vary according to students’ AI literacy. This integrated framework provides the theoretical basis for the direct, indirect, and conditional associations examined in the following hypotheses.

### 2.1 Generative AI technostress and learning engagement

Learning engagement reflects students’ behavioral participation, cognitive investment, emotional involvement, and social participation in learning processes (Fredricks et al., 2004; Zhoc et al., 2019). Engagement is shaped by both learning conditions and individual psychological resources (Li and Xue, 2023). When students experience elevated learning-related stress, cognitive and attentional resources may be directed toward managing the perceived demand rather than maintaining active involvement in academic activities (Frazier et al., 2019).

Generative AI can create technology-related demands because effective academic use requires students to understand system functions, formulate appropriate prompts, assess generated information, identify inaccuracies, and determine suitable boundaries for AI assistance (Kasneci et al., 2023). Students who perceive these requirements as difficult to manage may report greater uncertainty and lower perceived control over AI-supported learning activities. Existing technostress research has similarly associated stressful technology experiences with lower motivation and less favorable learning behaviors (Wang et al., 2020), while recent work suggests that difficulties in managing AI-related demands may also be associated with reduced involvement in learning activities.

Accordingly, higher levels of generative AI technostress are expected to be associated with lower levels of learning engagement.

*H1*: Generative AI technostress is negatively associated with university students’ learning engagement.

### 2.2 The mediating role of academic adaptability

Academic adaptability refers to students’ capacity to adjust their cognitive, behavioral, and emotional responses when encountering novelty, change, or uncertainty in academic environments (Martin, 2012). This capacity is relevant to GenAI-mediated learning because students may need to revise existing learning strategies, develop new approaches to information evaluation, and adjust their use of educational resources as AI-supported learning practices develop (Huo and Siau, 2024).

From the perspective of the transactional theory of stress and coping, greater perceived demands may be associated with greater difficulty in maintaining adaptive responses when students regard available coping resources as insufficient (Folkman, 2013). In the GenAI context, students reporting higher levels of technostress may also experience greater difficulty modifying learning strategies, regulating emotional responses, or maintaining flexible responses to changing AI-related academic requirements (Yin et al., 2024). This reasoning is consistent with evidence linking elevated stress with reduced adaptive functioning and with emerging research examining technostress and adaptability in changing learning environments (Martin, 2012; Ansari and Iqbal, 2025). Accordingly:

*H2*: Generative AI technostress is negatively associated with academic adaptability.

Academic adaptability is also expected to be positively associated with learning engagement. Students with stronger adaptability are better able to modify their learning strategies, behaviors, and emotional responses when academic circumstances change (Martin et al., 2013; Collie et al., 2017). In AI-mediated learning environments, this capacity may help students incorporate new tools into existing learning practices while maintaining attention to academic goals. Students who can adjust their learning strategies in response to technological change may therefore be more likely to sustain behavioral, cognitive, emotional, and social involvement in learning activities (Collie et al., 2017; Zhang and Xu, 2025; Xia et al., 2025). Accordingly:

*H3*: Academic adaptability is positively associated with university students’ learning engagement.

The preceding relationships also provide the basis for an indirect pathway. Generative AI technostress is expected to be negatively associated with academic adaptability, and academic adaptability is expected to be positively associated with learning engagement. Academic adaptability may therefore account for part of the statistical association between technostress and learning engagement. This proposed indirect relationship is consistent with the transactional theory of stress and coping because it positions students’ adjustment capacity between their perceived technology-related demands and their level of engagement in academic activities. Accordingly:

*H4*: Academic adaptability mediates the relationship between generative AI technostress and learning engagement.

### 2.3 The moderating role of AI literacy

Students differ in the resources available to understand and manage GenAI-related academic requirements. AI literacy refers to the knowledge, skills, and critical understanding required to recognize AI functions, use AI systems, evaluate AI-generated outputs, and make responsible decisions concerning AI use (Long and Magerko, 2020; Wang et al., 2023). These competencies may be relevant to how students interpret and respond to GenAI-related demands.

Students with lower AI literacy may have greater difficulty understanding AI capabilities and limitations or determining how generated information should be evaluated and used (Ng et al., 2021). Such uncertainty may be associated with greater difficulty maintaining adaptive learning responses when GenAI-related demands are perceived as stressful (Özbay, 2026). In contrast, students with stronger AI literacy may possess greater knowledge and practical competence for evaluating AI outputs and organizing their use of AI-supported learning resources (Bećirović et al., 2025).

This expectation is consistent with conservation of resources theory. AI literacy can be regarded as a personal resource that provides students with knowledge and competencies relevant to managing AI-related demands (Hobfoll, 1989; Hobfoll et al., 2018). Students with greater AI literacy may therefore show a weaker negative association between generative AI technostress and academic adaptability because they possess more resources for interpreting and managing AI-related requirements. Related research suggests that stronger AI competence can be associated with greater confidence in managing AI systems and more effective responses to AI-mediated learning requirements (Bewersdorff et al., 2025). Accordingly:

*H5*: AI literacy moderates the relationship between generative AI technostress and academic adaptability, such that the negative association is weaker among students with higher AI literacy.

### 2.4 Moderated mediation effect of AI literacy

The proposed mediation and moderation relationships can be combined into a conditional indirect model. Generative AI technostress is expected to be negatively associated with academic adaptability, which in turn is expected to be positively associated with learning engagement. At the same time, the strength of the association between technostress and adaptability is expected to vary according to students’ AI literacy.

Students with lower AI literacy may possess fewer AI-related cognitive and practical resources for interpreting system functions, evaluating generated outputs, and organizing appropriate AI-assisted learning strategies (Ng et al., 2021). Under these conditions, the negative association between generative AI technostress and academic adaptability may be stronger. Because academic adaptability is positively associated with learning engagement, a stronger negative technostress to adaptability association would correspond to a stronger negative indirect association between technostress and engagement.

Students with higher AI literacy may have greater resources for understanding AI functions, evaluating outputs, and regulating AI use. The negative association between technostress and adaptability is therefore expected to be weaker at higher levels of AI literacy, which would also correspond to a weaker negative indirect association between technostress and learning engagement through academic adaptability. This conditional pattern integrates the adjustment process described by the transactional theory of stress and coping with the resource-based differences proposed by conservation of resources theory:

*H6*: AI literacy moderates the indirect relationship between generative AI technostress and learning engagement through academic adaptability, such that the negative indirect association is stronger among students with lower AI literacy.

## 3 Method

### 3.1 Participants and data collection

A cross-sectional survey design was employed to investigate the relationships among generative AI technostress, academic adaptability, learning engagement, and AI literacy among university students. The target population consisted of undergraduate students with experience using generative AI tools for academic purposes. The accessible population comprised undergraduate students enrolled at Northeast Normal University during the data collection period.

Northeast Normal University was selected as the data collection site because the author was engaged in a collaborative research exchange at the university during the study period, which provided direct access to the student population and facilitated field data collection. A nonprobability convenience sampling strategy was used. Eligible undergraduate students were invited to participate through their academic counselors. The counselors distributed a Wenjuanxing survey link or QR code to students through student communication groups. Participation was voluntary, and students completed the online questionnaire independently using their own electronic devices.

Data were collected between March 1, 2026 and July 1, 2026 using Wenjuanxing, a widely used online survey platform in China. Before accessing the questionnaire, participants were informed about the purpose of the study, the voluntary nature of participation, the confidentiality of their responses, and their right to discontinue participation. Electronic informed consent was obtained before participants proceeded to the questionnaire. The study protocol was formally reviewed and approved by the Academic Ethics Committee of the College of Psychology at Northeast Normal University, Ethical Review Approval Number 2025093.

The questionnaire was administered in a fixed sequence for all participants. Participants first completed demographic and generative AI use questions, followed by the measures of generative AI technostress, academic adaptability, learning engagement, and AI literacy.

A total of 580 questionnaires were submitted. Before the substantive analyses, all responses were screened for completeness and response quality. Questionnaires were excluded when they contained substantial missing responses, when the total completion time was shorter than 60 s, or when the response pattern indicated highly repetitive or mechanically patterned responding. Patterned responses included selecting the same response option across all or nearly all scale items and completing long consecutive blocks of items using the same response category before systematically switching to another category. A completion time of less than 60 s was considered insufficient for participants to adequately read and respond to the full questionnaire. After applying these screening criteria, 51 questionnaires were excluded: 11 were incomplete, 18 were completed in less than 60 s, and 22 showed clear patterned responding. Consequently, 529 valid questionnaires were retained, resulting in a valid response rate of 91.21%. Although no formal *a priori* power analysis was conducted, the final sample of 529 participants was considered adequate for the proposed CFA, SEM, and bootstrap-based conditional process analyses given the complexity of the models and the stable estimation of model parameters.

The final sample included 203 female students and 326 male students. In terms of academic year, 199 participants were freshmen, 162 were sophomores, 105 were juniors, and 63 were seniors. The sample comprised 174 students from science disciplines, 185 from engineering, and 170 from humanities and liberal arts. Regarding weekly generative AI use duration, 88 participants reported 8 h or less per week, 157 reported 9 to 21 h, 170 reported 22 to 28 h, and 114 reported more than 28 h. The primary generative AI tools reported by participants included Doubao, DeepSeek, ChatGPT, and Gemini. Detailed sample characteristics are presented in Table 1.

**Table 1:** Demographic characteristics of the participants.

Characteristic	Category	*n*	%
Gender	Female	203	38.37
	Male	326	61.63
Academic year	Freshman	199	37.62
	Sophomore	162	30.62
	Junior	105	19.85
	Senior	63	11.91
Major type	Science	174	32.89
	Engineering	185	34.97
	Humanities and liberal arts	170	32.14
Weekly AI use duration	8 h or less	88	16.64
	9–21 h	157	29.68
	22–28 h	170	32.14
	More than 28 h	114	21.55
Primary AI tool	Doubao	232	43.86
	DeepSeek	155	29.30
	ChatGPT	88	16.64
	Gemini	54	10.21

### 3.2 Measures

All constructs were measured using established instruments. Participants responded to all measurement items using a five-point Likert scale ranging from 1 = strongly disagree to 5 = strongly agree. Adaptation was limited to wording necessary to make the instruments appropriate for the context of generative AI-supported learning, while the conceptual meaning and construct coverage of the original measures were retained.

Before the main data collection, the adapted questionnaire underwent two expert review for content validity. The experts examined whether the adapted wording remained consistent with the constructs represented in the original instruments and whether the items were clear and appropriate for university students using generative AI in academic contexts. Particular attention was given to items in which general references to technology or technology-enhanced learning were replaced with references to generative AI-supported learning. Revisions were made in response to the expert review where wording required clarification, while changes that would alter the substantive meaning of the original items were avoided.

#### 3.2.1 Generative AI technostress

Generative AI technostress (GT) was measured using a four-item measure adapted from the university students’ technostress scale developed by Wang et al. (2020). The original scale was developed for technology-enhanced learning environments and conceptualized technostress as the psychological pressure arising when students perceive a mismatch between their personal capabilities and technology-related learning demands or between their learning needs and the support provided by technology.

To adapt the measure to the present research context, general references to “technology” or “technology-enhanced learning” were replaced with references to “generative AI tools for learning, such as ChatGPT, DeepSeek, Gemini, or similar systems.” The adaptation changed the contextual referent of the items without changing their intended psychological meaning. The adapted items assessed difficulties in responding to AI-related learning requirements, adjusting established learning habits, investing additional time and effort, and managing disruptions associated with generative AI-supported learning. For example, one adapted item stated, “When using generative AI to complete learning tasks, I often find its functions or operations difficult to understand.” Higher scores indicated greater perceived technostress associated with the academic use of generative AI.

#### 3.2.2 Academic adaptability

Academic adaptability (AA) was measured using the 9-item Adaptability Scale applied by Martin et al. (2013), which was grounded in the adaptability framework proposed by Martin (2012). Academic adaptability refers to students’ capacity to adjust their cognitive, behavioral, and emotional responses constructively when confronted with novel, changing, or uncertain academic circumstances.

The items assessed students’ ability to revise their thinking and learning strategies, modify their behavior, and regulate their emotional responses when academic conditions changed. A representative item was, “When generative AI changes learning tasks or learning approaches, I am able to adjust my learning strategies in a timely manner.” In the present study, academic adaptability reflected students’ general capacity to maintain effective learning while responding to changes and uncertainties in contemporary academic environments. Higher scores indicated stronger academic adaptability.

In the measurement model, the nine academic adaptability items were aggregated into three item parcels. AAp1 comprised AA1, AA4, and AA7; AAp2 comprised AA2, AA5, and AA8; and AAp3 comprised AA3, AA6, and AA9. Each parcel score was calculated as the mean of its constituent items, and the three parcel scores were used as observed indicators of the latent academic adaptability construct in the CFA and SEM analyses.

#### 3.2.3 Learning engagement

Learning engagement (LE) was measured using the 28-item Higher Education Student Engagement Scale developed by Zhoc et al. (2019). The HESES was designed for higher education contexts and conceptualizes student engagement as a multidimensional construct comprising academic engagement, cognitive engagement, social engagement with peers, social engagement with teachers, and affective engagement. These five dimensions contain 8, 4, 4, 8, and 4 items, respectively.

The scale assessed students’ participation in academic activities, cognitive investment in learning tasks, interaction with peers and teachers, and affective involvement in learning. A representative item was, “I actively participate in course learning and course-related academic activities.” Higher scores indicated stronger overall learning engagement.

For the measurement model, the 28 learning engagement items were aggregated into five item parcels to reduce model complexity while retaining information from the full scale. The parcels were constructed using a systematic allocation procedure: LEp1 comprised LE1, LE6, LE11, LE16, LE21, and LE26; LEp2 comprised LE2, LE7, LE12, LE17, LE22, and LE27; LEp3 comprised LE3, LE8, LE13, LE18, LE23, and LE28; LEp4 comprised LE4, LE9, LE14, LE19, and LE24; and LEp5 comprised LE5, LE10, LE15, LE20, and LE25. Each parcel score was calculated as the mean of its constituent items. These five parcel scores were subsequently used as observed indicators of the latent learning engagement construct in the CFA and SEM analyses. The present study therefore focused on overall learning engagement rather than estimating separate structural effects for the five original HESES dimensions.

#### 3.2.4 AI literacy

AI literacy (AL) was measured using the 12-item Artificial Intelligence Literacy Scale developed by Wang et al. (2023). The scale assesses competence in understanding and interacting with artificial intelligence across four dimensions: awareness, use, evaluation, and ethics. Each dimension consists of three items.

The awareness dimension reflects students’ understanding of the presence, functions, and influence of AI technologies. The use dimension captures their ability to operate and apply AI tools to relevant tasks. The evaluation dimension assesses their capacity to critically examine AI systems and AI-generated outputs, while the ethics dimension reflects their understanding of responsible, fair, and appropriate AI use. A representative item was, “I understand the basic functions, scope of application, and major limitations of generative AI.” Higher scores indicated stronger AI literacy.

For the measurement model, the 12 AI literacy items were aggregated into four item parcels. ALp1 comprised AL1, AL5, and AL9; ALp2 comprised AL2, AL6, and AL10; ALp3 comprised AL3, AL7, and AL11; and ALp4 comprised AL4, AL8, and AL12. Each parcel score was calculated as the mean of its constituent items, and the four parcel scores were used as observed indicators of the latent AI literacy construct in the CFA. Although the original scale distinguishes awareness, use, evaluation, and ethics conceptually, the present structural analysis examined AI literacy as an overall latent construct rather than estimating separate effects for each subdimension.

### 3.3 Control variables

Gender, academic year, major type, and weekly AI use duration were included as control variables because these characteristics may be associated with students’ learning experiences or exposure to generative AI. Gender was entered as a binary indicator. Academic year was treated as a categorical variable, with freshman used as the reference category. Major type was represented by dummy variables, with science used as the reference category.

The categories for weekly AI use duration were predefined during questionnaire design rather than created *post hoc* based on the observed sample distribution. Weekly generative AI use was classified into four ranges: ≤8 h, 9–21 h, 22–28 h, and >28 h per week. The categorization was designed to distinguish different levels of AI use intensity while taking into account students’ potential use of generative AI for coursework, independent learning, literature searching, and other academic activities. Because these categories represented ranges of use rather than equal-interval measurements, weekly AI use duration was treated as a categorical variable rather than as a continuous predictor. The ≤8-h group was used as the reference category in the analyses.

The same set of control variables was included in the mediator and outcome equations used for the structural and conditional process analyses. Their coefficients were retained and reported in the Results section rather than being omitted from the model output.

### 3.4 Data analysis

All statistical analyses were conducted using R version 4.6.1. Confirmatory factor analysis (CFA) and structural equation modeling (SEM) were performed using the lavaan package version 0.7–2. Reliability analyses, including Cronbach’s alpha and McDonald’s omega, were conducted using the psych package version 2.6.5. Regression analyses and bootstrap procedures were implemented in R. Data preparation and management were conducted using the dplyr version 1.2.1 and tidyr version 1.3.2 packages.

The analyses followed a sequential procedure comprising data screening, descriptive analysis, measurement model assessment, common method bias assessment, structural model estimation, mediation analysis, moderation analysis, and moderated mediation analysis. All inferential tests were two-tailed, with the significance level set at *p* < 0.05 and confidence intervals reported at the 95% level.

Before the main analyses, the dataset was screened for completeness and response quality according to the criteria described in Section 3.1. Descriptive statistics were then calculated to summarize the study variables and participants’ AI use characteristics. Means and standard deviations were calculated for the principal continuous study variables, while frequencies and percentages were used to describe categorical variables. Pearson correlation coefficients were calculated to examine the bivariate associations among generative AI technostress, academic adaptability, learning engagement, and AI literacy.

The measurement model was assessed before the structural relationships were examined. CFA was conducted to evaluate whether the observed indicators adequately represented their hypothesized latent constructs. Model fit was evaluated using the chi square statistic, comparative fit index (CFI), Tucker Lewis index (TLI), root mean square error of approximation (RMSEA), and standardized root mean square residual (SRMR). The evaluation of model fit was based on the collective pattern of these indices rather than on any single criterion.

Internal consistency was evaluated using Cronbach’s alpha, McDonald’s omega, and composite reliability (CR). Convergent validity was assessed using standardized factor loadings and average variance extracted (AVE). Standardized factor loadings were reported for the measurement indicators to provide evidence regarding the contribution of each indicator to its intended construct. Discriminant validity was evaluated using both the Fornell and Larcker criterion and the heterotrait monotrait ratio (HTMT), with HTMT interpreted in accordance with Henseler et al. (2015).

To obtain a parsimonious measurement model while retaining information from the full item sets, item parceling was used for academic adaptability, learning engagement, and AI literacy. Academic adaptability was represented by three parcels derived from its nine items, learning engagement was represented by five parcels derived from its 28 items, and AI literacy was represented by four parcels derived from its 12 items. Parcel scores were calculated as the arithmetic mean of the items assigned to each parcel. Generative AI technostress contained only four items and was therefore modeled directly using its individual items as observed indicators. Accordingly, the four-factor CFA consisted of four individual-item indicators for generative AI technostress, three parcel indicators for academic adaptability, five parcel indicators for learning engagement, and four parcel indicators for AI literacy. The structural analyses examined the overall latent constructs rather than dimension-specific structural relationships.

Because the principal constructs were measured using a single self-report questionnaire at one time point, potential common method bias was examined. Following Podsakoff et al. (2003), Harman’s single-factor test was conducted. In addition, the theoretically hypothesized measurement model was compared with a single-factor CFA model. These procedures were used to evaluate whether the covariance among the study variables was dominated by a single common factor.

After establishing the adequacy of the measurement model, SEM was conducted to examine the hypothesized relationships among generative AI technostress, academic adaptability, and learning engagement. Gender, academic year, major type, and weekly AI use duration were included as control variables in the structural analyses. The coefficients associated with these control variables were also examined and reported in the Results section.

The mediating role of academic adaptability was examined using bootstrap estimation with 5,000 resamples. Direct, indirect, and total effects were estimated separately. An indirect effect was considered statistically significant when its 95% bootstrap confidence interval did not include zero.

The moderating role of AI literacy in the association between generative AI technostress and academic adaptability was examined using a regression model containing an interaction term. Generative AI technostress and AI literacy were mean-centered before the interaction term was calculated. The regression model included the centered main effects of generative AI technostress and AI literacy, their interaction term, and the control variables. A statistically significant interaction coefficient at *p* < 0.05 was interpreted as evidence of moderation. The interaction was further examined using simple slope analysis at one standard deviation below the mean, the mean, and one standard deviation above the mean of AI literacy.

Moderated mediation was evaluated by estimating whether the indirect association between generative AI technostress and learning engagement through academic adaptability varied according to AI literacy. Conditional indirect effects were estimated at low AI literacy, defined as one standard deviation below the mean, mean AI literacy, and high AI literacy, defined as one standard deviation above the mean. Bootstrap estimation with 5,000 resamples was used to generate 95% confidence intervals for the conditional indirect effects and the index of moderated mediation. Moderated mediation was considered supported when the 95% bootstrap confidence interval for the index of moderated mediation did not include zero.

## 4 Results

### 4.1 Correlations among variables

Prior to the structural analyses, Pearson correlations were calculated among the four principal study variables: generative AI technostress, academic adaptability, learning engagement, and AI literacy. As demonstrated in Table 2, the bivariate correlations among these primary variables align closely with the hypothesized directional relationships. Generative AI technostress displayed a negative statistical relationship with academic adaptability and learning engagement. Academic adaptability showed a positive association with learning engagement. AI literacy maintained a positive correlation with both academic adaptability and learning engagement, while demonstrating a comparatively weak relationship with generative AI technostress. All correlation coefficients among the variables remained well within conventional parameters, with none of the pairwise correlations exceeded 0.70, suggesting no indication of severe bivariate collinearity among the principal study variables.

**Table 2:** Correlation matrix for Pearson correlations among study variables.

Construct	GT	AA	LE	AL
GT	1.000			
AA	−0.379***	1.000		
LE	−0.452***	0.521***	1.000	
AL	−0.115**	0.399***	0.305***	1.000

*N* = 529. GT = Generative AI technostress; AA = Academic Adaptability; LE = Learning Engagement; AL = AI Literacy. **p < 0.01, ***p < 0.001.

### 4.2 Measurement model evaluation

A confirmatory factor analysis (CFA) was conducted to examine the adequacy of the four-factor measurement model comprising generative AI technostress, academic adaptability, learning engagement, and AI literacy. Generative AI technostress was represented by its four individual items, whereas academic adaptability, learning engagement, and AI literacy were represented by three, five, and four item parcels, respectively, as described in the Method section.

Reliability and convergent validity analyses were further conducted. As shown in Table 3, all constructs demonstrated satisfactory internal consistency. McDonald’s omega coefficients ranged from 0.828 to 0.906, exceeding the recommended value of 0.70. Composite reliability values ranged from 0.828 to 0.911, indicating satisfactory construct reliability.

**Table 3:** Reliability and convergent validity.

Construct	McDonald’s ω	CR	AVE	Cronbach’s α	Factor loadings
GT	0.828	0.828	0.547	0.828	0.719–0.753
AA	0.877	0.878	0.705	0.876	0.832–0.854
LE	0.906	0.911	0.673	0.906	0.782–0.852
AL	0.882	0.887	0.662	0.882	0.795–0.829

The average variance extracted (AVE) values ranged from 0.547 to 0.705, exceeding the recommended threshold of 0.50. These results support the convergent validity of the measurement model.

### 4.3 Discriminant validity assessment

Discriminant validity was verified through the application of the Fornell-Larcker criterion along with the assessment of the heterotrait-monotrait ratio (HTMT). As displayed within the matrix in Table 4, the square roots of the average variance extracted values for each construct exceeded the baseline correlation coefficients found between that construct and any alternative variables, satisfying the Fornell-Larcker requirements. To be exact, the square root of the average variance extracted was 0.740 for generative AI technostress, 0.840 for academic adaptability, 0.820 for learning engagement, and 0.814 for AI literacy.

**Table 4:** Discriminant validity evaluation using Fornell-Larcker and HTMT criteria.

Construct	GT	AA	LE	AL
GT	0.740			
AA	−0.442	0.840		
LE	−0.522	0.581	0.820	
AL	−0.134	0.453	0.338	0.814

Complementing these results, the heterotrait-monotrait ratio values generated across the variable pairings ranged safely between 0.134 and 0.582. These coefficients remain comfortably beneath the standard conservative threshold of 0.85, establishing that the four targeted constructs are empirically distinct and represent unique concepts within the broader research model.

### 4.4 Common method bias assessment

Because the study adopted a cross-sectional self-report questionnaire design, potential common method bias was examined.

Harman’s single-factor test was conducted. The first unrotated factor accounted for 39.36% of the total variance, which was below the commonly used threshold of 40% (Podsakoff et al., 2003). This result suggests that common method bias was unlikely to substantially influence the findings.

Second, confirmatory factor analysis was conducted to compare the hypothesized four-factor model with a single-factor model. The four-factor model demonstrated excellent fit: χ2 = 147.811, df = 98, CFI = 0.990, TLI = 0.987, RMSEA = 0.031, SRMR = 0.024. In contrast, the single-factor model showed substantially poorer fit: χ2 = 2193.259, df = 104, CFI = 0.568, TLI = 0.502, RMSEA = 0.195, SRMR = 0.153.

The substantial difference between the two models indicates that the measurement structure was unlikely to be explained by a single underlying factor. Taken together, these results did not indicate that a single common factor dominated the covariance among the study variables.

### 4.5 Structural model testing and hypothesis results

Following confirmation of the reliability and validity of the measurement framework, structural equation modeling was executed to investigate the hypothesized pathways linking generative AI technostress, academic adaptability, and learning engagement. To isolate these core relationships, individual characteristics including gender, academic year, major type, and weekly AI use duration were introduced into the model as control variables. The structural path coefficients and matching hypothesis outcomes are consolidated in Table 5.

**Table 5:** Structural model results of hypothesized relationships.

Path	*B*	SE	*β*	95% CI	*p*	Result
GT → AA	−0.384	0.045	−0.445	[−0.473, −0.296]	<0.001	Supported
AA → LE	0.352	0.040	0.434	[0.273, 0.430]	<0.001	Supported
GT → LE	−0.227	0.034	−0.325	[−0.293, −0.160]	<0.001	Supported

GT = Generative AI Technostress; AA = Academic Adaptability; LE = Learning Engagement. B = unstandardized coefficient; β = standardized coefficient.

As shown in Table 5, generative AI technostress was negatively associated with academic adaptability (*β* = −0.445, *p* < 0.001). This result indicates that students reporting higher levels of perceived AI-related technological stress tended to report lower levels of academic adaptability. Therefore, H2 was supported.

Academic adaptability was positively associated with learning engagement (*β* = 0.434, *p* < 0.001), suggesting that students with stronger adaptability tendencies tended to demonstrate higher levels of engagement in learning activities. Therefore, H3 was supported.

In addition, generative AI technostress showed a negative association with learning engagement (*β* = −0.325, *p* < 0.001), supporting H1. Taken together, these findings indicate that generative AI technostress was statistically related to both students’ adaptability experiences and their engagement levels.

### 4.6 Mediating role of academic adaptability

To examine whether the association between generative AI technostress and learning engagement was statistically indirect through academic adaptability, a bootstrap mediation analysis was conducted using 5,000 resamples. The direct, indirect, and total effect values calculated through this procedure are displayed in Table 6.

**Table 6:** Bootstrap mediation analysis of academic adaptability.

Effect	Estimate	SE	95% CI Lower	95% CI Upper	*p*	Proportion mediated
Indirect effect (GT → AA → LE)	−0.135	0.021	−0.180	−0.097	<0.001	37.32%
Direct effect (GT → LE)	−0.227	0.034	−0.293	−0.161	<0.001	
Total effect	−0.362	0.034	−0.430	−0.296	< 0.001	

The indirect association between generative AI technostress and learning engagement remained statistically significant after including academic adaptability (Estimate = −0.135, *p* < 0.001). Therefore, the results were consistent with academic adaptability partially mediating this relationship rather than fully accounting for it.

The indirect effect accounted for approximately 37.32% of the total association. This finding suggests that the relationship between generative AI technostress and learning engagement may be partly explained by differences in students’ academic adaptability. Thus, H4 was supported.

### 4.7 Moderating role of AI literacy

A moderation analysis was carried out to assess whether the association between generative AI technostress and academic adaptability varied across levels of AI literacy. In alignment with statistical recommendations, generative AI technostress and AI literacy scores were mean-centered prior to computing the product interaction term. The results derived from this interaction regression model are presented in Table 7.

**Table 7:** Moderating effect of AI literacy on the relationship between generative AI technostress and academic adaptability.

Predictor	*B*	*SE*	*t*	*p*
Constant	3.431	0.086	39.878	< 0.001
GT	−0.295	0.032	−9.097	< 0.001
AL	0.392	0.040	9.897	< 0.001
GT × AL	0.144	0.047	3.064	0.002

GT and AL were mean-centered before constructing the interaction term. Control variables included gender, academic year, major type, and weekly AI use duration.

The interaction term combining generative AI technostress and AI literacy emerged as statistically significant (*B* = 0.144, *t* = 3.064, *p* = 0.002). The positive direction of this coefficient indicates that individual AI literacy is associated with a weaker negative connection between generative AI technostress and student academic adaptability. More precisely, while heightened technostress correlates with lower levels of academic adaptability across the sample, this negative link is attenuated among students who possess a stronger command of AI literacy. This statistical trend suggests that digital and AI literacy competencies function as an important personal resource associated with students’ management of technological stress.

As shown in Table 8, the conditional association between generative AI technostress and academic adaptability was strongest at low AI literacy (*B* = −0.387, *p* < 0.001), weaker at the mean level (*B* = −0.295, *p* < 0.001), and weakest at high AI literacy (*B* = −0.203, *p* < 0.001).

**Table 8:** Simple slope analysis of AI literacy.

AI literacy level	*B*	SE	95% CI lower	95% CI upper	*p*
Low AI literacy (−1 SD)	−0.387	0.044	−0.473	−0.302	< 0.001
Mean AI literacy	−0.295	0.032	−0.359	−0.231	< 0.001
High AI literacy (+1 SD)	−0.203	0.045	−0.291	−0.114	< 0.001

The coefficients represent the conditional effect of generative AI technostress on academic adaptability at three levels of AI literacy.

**Figure 1:**
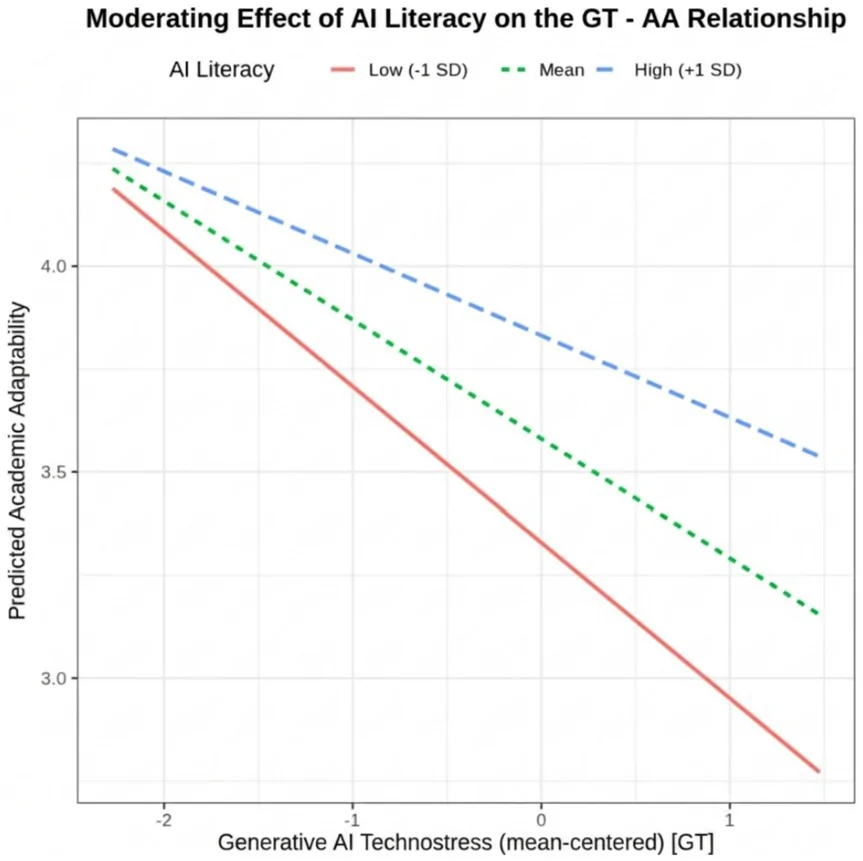
Moderating effect of AI literacy on the relationship between generative AI technostress and academic adaptability.

As illustrated in Figure 1, all three simple slopes were negative, but the slope became progressively flatter as AI literacy increased. Among students with low AI literacy (−1 SD), academic adaptability declined steeply as technostress rose, whereas among students with high AI literacy (+1 SD) the decline was markedly attenuated. This pattern visually confirms that AI literacy buffers the negative association between generative AI technostress and academic adaptability, supporting H5.

### 4.8 Moderated mediation analysis

To determine whether individual AI literacy conditions the indirect relationship linking generative AI technostress to learning engagement through academic adaptability, a conditional process evaluation was conducted using bootstrap estimation with 5,000 resamples. The conditional indirect effects computed across distinct baseline distributions of AI literacy are organized in Table 9.

**Table 9:** Conditional indirect effects across levels of AI literacy.

AI literacy level	Indirect effect	BootSE	95% CI lower	95% CI upper	*p*
Low AI literacy (−1 SD)	−0.118	0.017	−0.155	−0.086	< 0.001
Mean AI literacy	−0.090	0.013	−0.117	−0.065	< 0.001
High AI literacy (+1 SD)	−0.062	0.015	−0.093	−0.032	< 0.001
Index of moderated mediation	0.044	0.015	0.016	0.074	0.002

The results show that the indirect connection between generative AI technostress and student learning engagement via academic adaptability changes significantly depending on a student’s level of AI literacy. In particular, the negative indirect effect was strongest among university students reporting lower AI literacy capacities (Estimate = −0.118, 95% CI [−0.155, −0.086]) and became less pronounced as literacy scores increased. For individuals maintaining an average level of AI literacy, the indirect coefficient remained stable and significant (Estimate = −0.090, 95% CI [−0.117, −0.065]). Among students with high AI literacy levels, the negative indirect relationship was minimized but stayed statistically detectable (Estimate = −0.062, 95% CI [−0.093, −0.032]).

Because the 95% bootstrap confidence interval for the index of moderated mediation omitted zero (95% CI [0.016, 0.074]), H6 was supported. This pattern indicates that the negative indirect association between generative AI technostress and learning engagement through academic adaptability was stronger at lower levels of AI literacy and weaker at higher levels.

Table 10 shows that among the control variables, gender and academic year were significantly associated with academic adaptability. Male students showed slightly higher academic adaptability than female students (*B* = 0.154, *β* = 0.121, *p* = 0.007), and junior students showed higher academic adaptability than freshmen (*B* = 0.191, *β* = 0.123, *p* = 0.011). None of the remaining control-variable effects were statistically significant.

**Table 10:** Effects of control variables in the structural model.

Outcome	Predictor	B	SE	*β*	*p*	95% CI
AA	Male vs. Female	0.154	0.057	0.121	0.007	[0.043, 0.266]
AA	Sophomore vs. Freshman	0.046	0.062	0.034	0.460	[−0.075, 0.166]
AA	Junior vs. Freshman	0.191	0.075	0.123	0.011	[0.043, 0.339]
AA	Senior vs. Freshman	−0.007	0.094	−0.004	0.941	[−0.191, 0.177]
AA	Engineering vs. Science	−0.013	0.064	−0.010	0.833	[−0.139, 0.112]
AA	Humanities vs. Science	−0.060	0.064	−0.045	0.349	[−0.185, 0.065]
AA	9–21 h vs. ≤ 8 h	0.027	0.074	0.020	0.718	[−0.118, 0.172]
AA	22–28 h vs. ≤ 8 h	0.026	0.078	0.020	0.736	[−0.127, 0.180]
AA	>28 h vs. ≤ 8 h	0.039	0.083	0.026	0.639	[−0.123, 0.201]
LE	Male vs. Female	0.037	0.038	0.036	0.338	[−0.039, 0.112]
LE	Sophomore vs. Freshman	−0.032	0.043	−0.029	0.461	[−0.117, 0.053]
LE	Junior vs. Freshman	−0.059	0.049	−0.047	0.222	[−0.155, 0.036]
LE	Senior vs. Freshman	−0.079	0.073	−0.051	0.280	[−0.221, 0.064]
LE	Engineering vs. Science	−0.005	0.045	−0.005	0.904	[−0.093, 0.083]
LE	Humanities vs. Science	−0.003	0.045	−0.003	0.945	[−0.091, 0.085]
LE	9–21 h vs. ≤ 8 h	0.053	0.055	0.048	0.338	[−0.055, 0.161]
LE	22–28 h vs. ≤ 8 h	−0.029	0.057	−0.027	0.611	[−0.141, 0.083]
LE	>28 h vs. ≤ 8 h	0.021	0.059	0.017	0.723	[−0.095, 0.137]

Reference categories were female for gender, freshman for academic year, science for major type, and ≤8 h for weekly AI use duration.

## 5 Discussion

### 5.1 Discussion of findings

The findings showed that generative AI technostress was negatively associated with both learning engagement and academic adaptability. This pattern is consistent with previous research showing that technology-related stress is associated with less favorable motivational, behavioral, and psychological outcomes in educational settings (Wang et al., 2020; Frazier et al., 2019), as well as with Saleem et al. (2024), who found that technostress was negatively associated with the quality of online learning among university students. It also accords with recent work suggesting that difficulty managing AI-related demands may be associated with lower involvement in learning activities. However, Lallmahomed (2025) reported a more differentiated pattern among university students in Mauritius, finding that hindrance techno-stressors were negatively associated with student engagement, whereas challenge techno-stressors were positively associated with engagement. This divergence may reflect differences in the conceptualization of technostress and the technological context, suggesting that technology-related demands are not uniformly detrimental when some demands are appraised as manageable challenges rather than hindrances. In the present study, students who reported greater difficulty understanding, evaluating, or appropriately using generative AI also tended to report lower engagement and adaptability, possibly because the uncertainty and cognitive demands associated with GenAI compete with the attention and psychological resources required for sustained learning.

Academic adaptability was positively associated with learning engagement and partially accounted for the association between generative AI technostress and learning engagement. This finding is consistent with previous research linking adaptability with stronger academic functioning and engagement under changing educational conditions (Martin et al., 2013; Collie et al., 2017), and with studies emphasizing the importance of adaptability in technology-rich learning environments (Zhang and Xu, 2025; Xia et al., 2025). The mediation result suggests that part of the association between GenAI technostress and engagement is related to students’ capacity to adjust their cognition, behavior, and emotions when learning conditions change. Academic adaptability therefore provides a plausible psychological explanation for why students reporting greater GenAI-related stress also tend to report lower engagement. Given the cross-sectional design, however, this finding should be interpreted as an indirect association rather than a causal process.

AI literacy moderated the negative association between generative AI technostress and academic adaptability, with a weaker negative association among students reporting higher AI literacy. This pattern is consistent with research suggesting that stronger AI-related knowledge and competence are associated with greater ability to understand AI functions, evaluate outputs, and manage AI-supported learning activities (Ng et al., 2021; Bećirović et al., 2025; Bewersdorff et al., 2025). Students with greater AI literacy may have clearer expectations about AI capabilities and limitations and may therefore experience less uncertainty when deciding how to use AI in academic work.

The moderated mediation analysis further showed that the negative indirect association between generative AI technostress and learning engagement through academic adaptability was stronger at lower levels of AI literacy and weaker at higher levels. This suggests that the relationship between technostress and engagement varies according to both students’ adaptive capacity and their AI-related competence. Rather than operating independently, academic adaptability and AI literacy appear to represent complementary elements of students’ adjustment to GenAI-mediated learning.

### 5.2 Theoretical implications

The findings provide several implications for the application of the transactional theory of stress and coping to GenAI-mediated learning. The negative associations of generative AI technostress with academic adaptability and learning engagement support the theory’s proposition that individuals’ responses to environmental demands depend on appraisal and adjustment processes rather than on the objective presence of the demand alone (Folkman, 2013). More importantly, the mediation finding extends this perspective by identifying academic adaptability as a specific adjustment-related construct through which perceived AI-related demands are associated with learning engagement. The findings therefore help distinguish between the experience of technological demands and students’ capacity to adjust to those demands in AI-mediated learning environments.

The results also refine the application of conservation of resources theory. AI literacy has generally been conceptualized as knowledge, practical competence, evaluation, and ethical understanding (Long and Magerko, 2020; Wang et al., 2023). The moderation finding suggests that these competencies may also function as context-relevant personal resources because the negative association between technostress and adaptability was weaker among students with higher AI literacy. This extends COR theory to AI-mediated higher education by indicating that the protective value of a resource may depend partly on its relevance to the specific demands being encountered.

Taken together, the findings provide an integrated theoretical account of the proposed model. The transactional theory of stress and coping explains the technostress–adaptability–engagement pathway, whereas conservation of resources theory helps explain why the technostress–adaptability association varies according to AI literacy. The moderated mediation result supports this combined interpretation by showing that the indirect association through adaptability differs across levels of the proposed personal resource. The findings therefore suggest that process-oriented and resource-oriented explanations can be jointly applied to students’ responses to emerging educational technologies.

At the same time, the findings also indicate limits to this explanation. The direct association between technostress and learning engagement remained significant after academic adaptability was included, and the negative association between technostress and adaptability remained significant even at higher levels of AI literacy. Academic adaptability and AI literacy therefore explain only part of the observed relationships. Other psychological resources, institutional conditions, learning demands, and forms of AI experience may also be relevant, which defines an important boundary of the present theoretical model.

### 5.3 Practical implications

The negative association between generative AI technostress and learning engagement suggests that university management and lecturers should consider students’ experiences of AI-related demands alongside the availability of AI tools. Institutional approaches to GenAI should therefore include guidance on evaluating generated information, identifying unreliable outputs, and determining appropriate forms of AI assistance. Such guidance may help reduce uncertainty surrounding academic AI use and provide students with clearer expectations regarding how GenAI can be incorporated into learning activities.

The positive association between academic adaptability and learning engagement, together with its mediating role, has implications for lecturers, curriculum developers, and student-support services. Support for GenAI use should not focus only on technical competence. Learning activities can also provide opportunities for students to revise strategies, compare AI-assisted and independent approaches, evaluate and correct AI-generated responses, and reflect on how their learning practices need to change when using AI. These activities are closely related to the cognitive and behavioral components of adaptability and may help students develop more flexible responses to changing learning conditions.

The moderation finding identifies AI literacy as another relevant area for institutional support. Curriculum developers and lecturers may place greater emphasis on students’ understanding of AI capabilities and limitations, evaluation of generated content, responsible use, and appropriate reliance on AI systems. Because the negative association between technostress and adaptability was stronger among students with lower AI literacy, support may be particularly relevant for students with limited previous exposure to generative AI. This does not imply that higher AI literacy eliminates technostress, but it suggests that AI-related competence is associated with a less pronounced relationship between technostress and reduced adaptability.

The moderated mediation finding further suggests that AI literacy and academic adaptability can be considered together when designing student support. Rather than treating AI literacy as a one-time technical orientation, universities may integrate AI literacy development with opportunities for repeated academic practice and reflection. University management can provide institutional guidance, curriculum developers can embed AI-related competencies within coursework, lecturers can provide discipline-relevant practice, and student-support services can offer additional assistance to students who experience difficulty adapting to AI-supported learning. Such differentiated support is more closely aligned with the observed variation in the associations among technostress, adaptability, AI literacy, and engagement.

### 5.4 Limitations and future research

First, this study adopted a cross-sectional research design. Therefore, the observed relationships among generative AI technostress, academic adaptability, learning engagement, and AI literacy should not be interpreted as evidence of causal relationships. Future research may employ longitudinal designs or experimental approaches to examine how these relationships develop over time.

Second, all variables were measured through self-reported questionnaires, which may introduce response bias. Although statistical procedures were used to assess common method bias, future studies could combine self-report measures with behavioral indicators such as AI usage records, learning activity data, or instructor evaluations. The measures were also administered in a fixed order rather than a randomized order, which may have introduced order effects or response fatigue. Future research could randomize measurement blocks or use counterbalanced questionnaire versions. In addition, although the adapted questionnaire underwent expert review for content validity, a separate formal pilot study was not conducted. Future studies could use an independent pilot sample to further examine item clarity and response processes before full-scale administration.

Third, the sample was drawn from a single university in China. The observed relationships should therefore be interpreted within this institutional and cultural context. Future studies may examine whether similar patterns are observed across universities, countries, academic disciplines, and educational systems.

Fourth, the study focused on academic adaptability as the principal explanatory mechanism and AI literacy as the moderating resource. Other psychological and contextual factors may also be relevant, including AI anxiety, self-efficacy, perceived autonomy, learning strategies, institutional guidance, and previous AI experience. Examining these factors may provide a broader account of variation in students’ responses to GenAI-mediated learning environments.

## 6 Conclusion

This study examined the associations among generative AI technostress, academic adaptability, learning engagement, and AI literacy among undergraduate students. Generative AI technostress was negatively associated with both academic adaptability and learning engagement, whereas academic adaptability was positively associated with learning engagement. Academic adaptability partially accounted for the association between generative AI technostress and learning engagement. AI literacy moderated the association between technostress and academic adaptability, with a weaker negative association observed at higher levels of AI literacy. The conditional indirect association between generative AI technostress and learning engagement through academic adaptability was also stronger among students with lower AI literacy.

These findings support an integrated interpretation of the transactional theory of stress and coping and conservation of resources theory in GenAI-mediated higher education. Academic adaptability represents a relevant adjustment process associated with students’ responses to AI-related demands, while AI literacy represents a context-relevant personal resource associated with variation in this process. From a practical perspective, the findings suggest that universities should consider students’ adaptability and AI literacy alongside technological access when developing GenAI-related teaching practices, curriculum support, and student services. Given the cross-sectional design, these conclusions concern statistical associations and should not be interpreted as evidence of causal effects.

## Data Availability

The raw data supporting the conclusions of this article will be made available by the authors, without undue reservation.
